# Physiological, Perceptual, and Performance Responses to the 2-Week Block of High- versus Low-Intensity Endurance Training

**DOI:** 10.1249/MSS.0000000000002861

**Published:** 2022-01-24

**Authors:** OLLI-PEKKA NUUTTILA, ARI NUMMELA, HEIKKI KYRÖLÄINEN, JARI LAUKKANEN, KEIJO HÄKKINEN

**Affiliations:** 1 Faculty of Sport and Health Sciences, University of Jyväskylä, Jyväskylä, FINLAND; 2 KIHU – Research Institute for Olympic Sports, Jyväskylä, FINLAND; 3 Institute of Public Health and Clinical Nutrition, University of Eastern Finland, Kuopio, FINLAND; 4 Department of Internal Medicine, Central Finland Health Care District, Jyväskylä, FINLAND

**Keywords:** BLOCK PERIODIZATION, RUNNING, ENDURANCE PERFORMANCE, HEART RATE VARIABILITY, NOREPINEPHRINE, MUSCLE SORENESS

## Abstract

**Purpose:**

This study examined the physiological, perceptual, and performance responses to a 2-wk block of increased training load and compared whether responses differ between high-intensity interval (HIIT) and low-intensity training (LIT).

**Methods:**

Thirty recreationally trained males and females performed a 2-wk block of 10 HIIT sessions (INT, *n* = 15) or 70% increased volume of LIT (VOL, *n* = 15). Running time in the 3000 m and basal serum and urine hormone concentrations were measured before (T_1_) and after the block (T_2_), and after a recovery week (T_3_). In addition, weekly averages of nocturnal heart rate variability (HRV) and perceived recovery were compared with the baseline.

**Results:**

Both groups improved their running time in the 3000 m from T_1_ to T_2_ (INT = −1.8% ± 1.6%, *P* = 0.003; VOL = −1.4% ± 1.7%, *P* = 0.017) and from T_1_ to T_3_ (INT = −2.5% ± 1.6%, *P* < 0.001; VOL = −2.2% ± 1.9%, *P* = 0.001). Resting norepinephrine concentration increased in INT from T_1_ to T_2_ (*P* = 0.01) and remained elevated at T_3_ (*P* = 0.018). The change in HRV from the baseline was different between the groups during the first week (INT = −1.0% ± 2.0% vs VOL = 1.8% ± 3.2%, *P* = 0.008). Muscle soreness increased only in INT (*P* < 0.001), and the change was different compared with VOL across the block and recovery weeks (*P* < 0.05).

**Conclusions:**

HIIT and LIT blocks increased endurance performance in a short period. Although both protocols seemed to be tolerable for recreational athletes, a HIIT block may induce some negative responses such as increased muscle soreness and decreased parasympathetic activity.

## Abstract 2

Supplemental digital content is available in the text.

The aim of the athletic training process is to produce adequate stimuli that would lead to positive training adaptations. In endurance training, the variables that are typically modified to induce desirable responses are the intensity, duration, and frequency of training (1). In long-term periodization, it seems necessary to perform high volumes of endurance training at low intensity (1). However, in short-term periodization, block periodization—altering focus between volume and intensity (2)—or polarized periodization—mixing low- and high-intensity training (3)—have both been suggested to be the most favorable training organization methods.

Block periodization protocols have typically focused on high-intensity interval training (HIIT) consisting of 1- to 3-wk microcycles of multiple weekly or even daily high-intensity sessions (4). On the other hand, studies examining the effects of high-volume microcycles have most often included overload periods increasing both low- and high-intensity training volume (5). The length of the periods has varied predominantly between 2 and 6 wk, during which training volume has been increased by 30%–100% from the volume previously used by an individual (6–9). High-intensity and high-volume endurance training periods have mainly been studied separately, but possible differences in the physiological, perceptual and performance responses are not well established.

When there is a substantial increase in training load from the previous load, there is also an increased risk of injuries (10) and maladaptation or overreaching (5). To avoid such consequences, it would be critical to detect early signs that may predict compromised training adaptations. Monitoring of training and recovery typically consists of regular assessments of physiological, perceptual, or performance-related markers that are estimated to provide valuable information about the recovery and training state of an athlete (11). On one end of the monitoring tool spectrum are extensive laboratory tests, such as hormonal or biochemical examinations from blood or urine (6,12), whereas perceptual markers such as subjective surveys (13,14) or session RPE (15) represent the other end of the spectrum. In addition, noninvasive assessments of physiological markers, like heart rate variability (HRV) recordings at rest (16), heart rate (HR) during exercise (17), and performance-related markers such as various jumping tests (18), could be used in monitoring. The purpose of the monitoring process is to follow whether an athlete is adapting to the stimulus as expected and to influence decisions for the forthcoming training load (18) or session intensity (16,17).

Although monitoring has clear advantages during the training process, previous studies have disclosed several contradictions and limitations, especially regarding responses of physiological markers. In the case of submaximal HR and resting HRV, it is a well-known dilemma that a similar type of response may be observed after both a positive training adaptation and in the state of parasympathetic hyperactivity, which is associated with a decrease in maximal performance (8,19). Furthermore, plasma volume expansion may, at least acutely, affect HRV (20), regardless of the recovery state. Resting levels of catecholamines, which correlate with sympathetic nervous system activity, have previously been reported as unchanged in female endurance athletes (7) and male triathletes (21) but decreased in well-trained runners (6) after an intensified training period. In the same studies, acute responses of catecholamines to maximal exercise have also varied between unchanged (6,21) and decreased (7,21) after a period of intensified training. It has been suggested that, in general, hormonal responses to maximal exercise may be altered more than resting levels in the overtraining state (12), making regular hormonal assessments in athletes rather difficult. Acknowledging these challenges with physiological markers, subjective estimations of recovery may provide valuable “triangulating” information that improves interpretation of athlete status during training (13,14) and helps to contextualize complicated physiological changes (19).

The aim of the present study was to examine the physiological, perceptual, and performance responses to blocks of increased training load, and to compare whether these responses would differ between high-volume low-intensity training (LIT) and HIIT periods in recreationally trained male and female participants. Another aim was to explore whether training adaptation would be associated with the responses of the monitoring variables. We hypothesized that both types of training blocks would improve endurance performance after the recovery week but induce acute fatigue immediately after the training period, observed as decreased or unchanged performance and impaired perceptual recovery (8,19).

## METHODS

### Participants

A total of 40 recreationally endurance-trained male and female runners were recruited to participate voluntarily in the study. Participants were 20–45 yr old, healthy, and experienced in regular running training (>4 times per week). A cardiologist checked electrocardiography of all potential participants before the final acceptance to participate. One participant dropped out before any measurement because of difficulties with the timetable. In addition, six participants dropped out because of sicknesses (*n* = 2) or injuries (*n* = 4) that occurred during the preparatory period or at the beginning of the training period. From the participants that finished the whole study period, one participant was excluded from the final analysis because of insufficient training adherence (<90%/main sessions), and two participants for not following the training instructions during the preparatory or recovery periods. Baseline characteristics of the participants that were included in the final analysis (*n* = 30) are presented in Table 1. All participants gave their written consent to participate, and the study protocol was approved by the ethics committee of the University of Jyväskylä.

**TABLE 1:** Mean ± SD baseline characteristics of the participants.

	INT (*n* = 15)	VOL (*n* = 15)
Sex (male/female)	9/6	9/6
Age (yr)	33 ± 7	37 ± 7
Height (cm)	172 ± 10	174 ± 11
Body mass (kg)	72 ± 14	71 ± 13
v_LT1_ (km·h^−1^)	10.8 ± 1.2	10.7 ± 1.4
v_LT2_ (km·h^−1^)	13.3 ± 1.7	13.0 ± 1.6
v_max_ (km·h^−1^)	16.6 ± 1.8	16.4 ± 1.8
V̇O_2max_ (mL·kg^−1^·min^−1^)	50.4 ± 6.9	49.7 ± 6.4
3000 m (min:s)	12:29 ± 1:36	12:34 ± 1:35

INT, interval group; VOL, volume group; v_LT1_, running speed at the first lactate threshold; v_LT2_, running speed at the second lactate threshold; v_max,_ maximal speed of the incremental treadmill test; V̇O_2max_, maximal oxygen uptake. Baseline characteristics were measured before the preparatory period (T_0_).

### Study Protocol

The study consisted of four separate phases similar to the protocol used by Le Meur et al. (8): a 2-wk preparatory period (phase 1), the first recovery week (phase 2), a 2-wk training period (phase 3), and the second recovery week (phase 4). Participants were advised to continue their regular training in terms of volume during the preparatory period and to decrease training volume by 50% in the following recovery week. To ensure a similar training intensity distribution before the training intervention, participants were asked to train below the first lactate threshold, excluding one HIIT session (6 × 3 min), which was performed to familiarize participants with the interval protocol. At the end of the preparatory period, participants were matched into pairs based on sex, 3000-m performance, v_max_, and baseline HRV, and divided into the interval group (INT) or volume group (VOL). During the 2-wk training period, the INT group performed a total of 10, 6 × 3-min HIIT sessions (5 sessions per week), whereas the VOL group increased their low-intensity running volume (h) by 70%. Proper training load for the HIIT and VOL protocols was estimated based on previous studies examining HIIT shock microcycles (4) or volume-based overload periods (6–8). After the 2-wk training period, a similar recovery week as the first was prescribed. Performance in the 3000 m and countermovement jump (CMJ) were measured, and fasting blood and urine samples were taken and analyzed before the preparatory period (T_0_), in the middle of the first recovery week (T_1_), 1 d after the intensified training period (T_2_), and after the second recovery week (T_3_). An incremental treadmill test was performed once in the same week as the other T_0_ tests to analyze lactate thresholds (LT1 and LT2) and individual training intensity zones among the participants. A day of rest was always prescribed before testing days. Training and recovery were monitored with multiple markers throughout the study.

### Training Protocol

Both groups had five main sessions per week, which were supervised and performed individually at the same time of the day (±2 h) during the morning or afternoon and at the same outdoor road/track, which was tight gravel (INT) or about 50/50 combination of gravel and asphalt (VOL). The INT group performed all the sessions as 6 × 3-min intervals, whereas the VOL group performed only low-intensity sessions below the first lactate threshold. If participants performed more than five sessions during the preparatory period, these sessions were also incorporated into the training period as low-intensity training with the same duration (INT) or with increased duration (VOL) to match the requirement of the volume increment. In case participants were accustomed to alternative endurance training modes such as cycling, these modes were incorporated as part of the additional sessions with similar proportion to the preparatory period.

#### Interval session

HIIT session was a 6 × 3-min interval with 2-min active recovery (walking). Intervals were performed at the maximal sustainable effort (22). Before the session, a 15-min warm-up, including three 30-s accelerations to the target speed, was performed. After the session, a 10-min cooldown was prescribed. Average running speed and HR were calculated separately for each interval and for the entire session, and a session RPE score was reported after each session (15).

#### Low-intensity sessions

The VOL group performed four similar basic sessions (85%–95% HR of the LT1) and one long-distance session (75%–90% HR of the LT1) in a week. The aim was to increase the duration of running sessions compared with preparatory period. The duration of these sessions was individually scaled based on the training during the preparatory period. The basic session was planned to be approximately 1.50× the average session duration during the preparatory period (1:22 ± 0:10 h:min), whereas the long-distance session was 1.66× the duration of the basic session (2:16 ± 0:16 h:min). Average running speed, average HR, and HR running speed index (HR-RS index) (23) were calculated from all supervised sessions. In addition, session RPE was estimated after all sessions (15).

### Performance Tests

An incremental treadmill test was performed on a treadmill (Telineyhtymä Oy, Kotka, Finland). The starting speed was set to 7 or 8 km·h^−1^ for women and 8 or 9 km·h^−1^ for men. Three-minute stages and speed increments of 1 km·h^−1^ were used. After each stage, the treadmill was stopped, and participants stood still for the fingertip blood lactate samples, which took approximately 15–20 s. Incline was kept constant at 0.5° throughout the test. Oxygen consumption was measured breath by breath (Jaeger VyntusTM CPX, CareFusion Germany 234 GmbH, Hoechberg, Germany), and HR was monitored with Garmin Forerunner 245 M (Garmin Ltd., Schaffhausen, Switzerland). Maximal oxygen uptake (V̇O_2max_, mL·kg^−1^·min^−1^) was defined as the highest 60 s average of oxygen consumption. Maximal running speed (v_max_) of the test was defined as the highest completed speed, or if the stage was not finished, as a speed of the last completed stage (km·h^−1^) + (running time (s) of the unfinished stage – 30 s)/(180–30 s) × 1 km·h^−1^. The first lactate threshold (LT1) and the second lactate threshold (LT2) were determined based on lactate values during the test. The LT1 was set at 0.3 mmol·L^−1^ above the lowest lactate value and LT2 at the intersection point between 1) a linear model between LT1 and the next lactate point and 2) a linear model for the lactate points measured after the point when La increased at least 0.8 mmol·L^−1^ for the first time. The same treadmill and lactate threshold estimation protocols have been used in previous studies (16,24,25).

The 3000-m running test was performed on a 200-m indoor track. Before the test, 15-min low-intensity warm-up was performed, including 3 × 20–30 s accelerations to target pace at the latter part of the warm-up. Verbal encouragement and split times (1000 m, 2000 m) were given for all participants during the test. The test was run in small groups (maximum seven persons). All test attempts were performed individually at the same time of the day (±2 h) during the afternoon or evening.

The CMJ test was performed before supervised sessions and before the 3000-m running tests. In the test, participants performed three maximal attempts on a contact mat with a 1-min recovery. The test was performed after a standardized warm-up, including a short jog (~3 min) and two sets of different kinds of squats (half squat, lunge, and squat jump). Jump height (h) was calculated based on the measured flight time with the following formula: *h* = *g* × *t*^2^ × 8^−1^, where *t* is the recorded flight time in seconds and *g* is the acceleration due to gravity (9.81 m·s^−2^) (26). The highest jump height (cm) was used in the data analysis.

### Blood and Urine Samples

Fasting blood samples were taken after 12 h of fasting and individually at the same time of the day (7:00–9:15 am). Blood samples were taken in a seated position from the antecubital vein into 6 mL serum tubes using standard laboratory procedures. Whole blood was centrifuged at 2250*g* (Megafuge 1.0 R, Heraeus, Hanau, Germany) for 10 min, and the separated serum was removed and frozen at −20 C until analysis. Serum cortisol concentration was analyzed with a chemical luminescence technique (Immulite 2000 XPi, Siemens, New York City, NY). The sensitivity of the cortisol assay was 5.5 nmol·L^−1^, and the intra-assay coefficient of variation was 5.3%. Free testosterone concentration was analyzed with ELISA (DYNEX DS 2 ELISA processing system, DYNEX Technologies, Chantilly, VA). The sensitivity of the free testosterone assay was 0.6 pmol·L^−1^, and the intra-assay coefficient of variation was 6.0%. Serum creatine kinase activity was analyzed with Indiko Plus Clinical Chemistry Analyzer (Thermo Fisher Scientific, Vantaa, Finland). The sensitivity of the creatine kinase assay was 2.2 U·L^−1^, and the intra-assay coefficient of variation was 0.9%. Hemoglobin and hematocrit were analyzed with an automated hematology analyzer (Sysmex XP-300TM, Sysmex Inc., Kobe, Japan). Plasma volume was estimated from the obtained hematocrit and hemoglobin values based on the equation of Dill and Costill (27).

Urine sample collection was performed between 1900 and 0700 h during the night before fasting samples were taken. Participants were asked to document the accurate starting and ending times of the collection. After bringing the sample to the laboratory, the urine volume was determined. For the analysis of norepinephrine, a 10-mL sample was frozen at −20°C. The concentrations of hormones in the sample were assessed by the liquid chromatography (HPLC) method (Labor Dr. Kramer & Kollegen, Geesthacht, Germany). The intra-assay coefficient of variation for the norepinephrine was 2.0%. Because of slight differences in collection times, the concentration of hormones in the urine sample was multiplied by the volume of the whole urine, then divided by the collection time in hours, and multiplied by 12 to represent a similar 12-h collection time for all participants similar to Hynynen et al. (28).

### Training and Recovery Monitoring

Participants wore an HR monitor (Garmin Forerunner 245 M) during all endurance training sessions. HR and GPS data (distance covered, running speed) were analyzed from all sessions. Training intensity distribution was analyzed with a time in zone model (HR_zone1_, HR < LT1; HR_zone2_, HR = LT1–LT2; HR_zone3_, HR > LT2). Participants wrote in the training log basic information of each session performed and estimated session RPE on a 0–10 scale (15).

Nocturnal HR and HRV were recorded with the Firstbeat Bodyguard 2 device (Firstbeat Technologies LTD, Jyväskylä, Finland). Participants were advised to start recording when going to sleep and stop the recording right after awakening. Recordings were performed every night starting from the first recovery week. Recorded RR intervals were edited by an artifact detection filter within the Firstbeat Sports software, which excluded all falsely detected, missed, and premature heartbeats. If the error percentage representing the number of corrected interbeat intervals shown by the software was higher than 33%, recordings were excluded from the analysis, as suggested by Vesterinen et al. (24). One participant in the VOL group had a high amount of erroneous data (error percentage >33% more than 50% of the recorded nights) and was excluded from the nocturnal analysis. Average HR, natural logarithm of high-frequency power (lnHF), and natural logarithm of the root-mean-square of the successive differences (lnRMSSD) were analyzed from the sleep period of 0030–0430 h after going to bed. High intraclass correlation coefficients of 0.97 and 0.91 have been reported in HR and HF, respectively, when 4-h averages have been compared between two consecutive nights after a similar training day (29). Weekly average values were used as suggested by Le Meur et al. (8): Pre, recovery week preceding the training period; Week1, first week of the training period; Week2, second week of the training period; Week3, recovery week after the training period.

Participants filled out daily questionnaires on a 0–10 visual analog scale (VAS) regarding estimated readiness to train, sleep quality of the previous night, general fatigue, muscle soreness of lower extremities, and perceived stressfulness during the day. Questionnaires were modified from the previous studies (13,14). Results were averaged similarly to nocturnal HR and HRV results.

### Statistical Analysis

Results are presented as mean ± SD. Before performing the final analysis, we determined if the magnitude of changes in the main variables differed between sexes (Kruskal–Wallis test). No significant differences were found; thus, female and male participants were analyzed in combined groups. The normality of the data was assessed with the Shapiro–Wilk test. To examine the main effects (time, group) and their interaction (time–group) in the monitoring variables (Pre, Week1, Week2, and Week3), performance or laboratory tests (T_0,_ T_1,_ T_2,_ T_3_), and training characteristics of the main sessions (1st vs 2nd–10th sessions), repeated-measures ANOVA was applied. In the case of a significant main effect or interaction, a Bonferroni *post hoc* test was used for within-group comparisons and simple contrasts for between-group comparisons. Training characteristics (frequency, volume, running kilometers, and training intensity distribution), creatine kinase, and free testosterone results were not normally distributed; thus, the Wilcoxon signed rank test was used for comparisons between time points and the Mann–Whitney *U*-test for between-group comparisons, with Bonferroni correction (*P* values multiplied by the number of comparisons). To examine the magnitude of observed changes, the effect size (ES) of within-group absolute differences was calculated as Cohen’s *d* for the main variables, and after nonparametric tests by the following formula: ES = *Z*(√*n*)^−1^, where *Z* is the *z*-score, and *n* is are the number of observations on which *Z* is based. The magnitude of changes was categorized as <0.2 trivial, 0.2–0.5 small, 0.5–0.8 moderate, and >0.8 large. In addition, the Pearson correlation coefficient was used to analyze relationships between the monitoring variables (absolute values at Week2, changes from Pre to Week2 or changes from 1st session to 10th session) and changes in the 3000-m running speed (km·h^−1^, T_1_–T_2_ Δ%, T_1_–T_3_ Δ%). The statistical significance level was set to *P* < 0.05. Analyses were performed with Microsoft Excel 2010 (Microsoft Corporation, WA) and IBM SPSS Statistics version 26 programs (SPSS Inc, Chicago, IL).

## RESULTS

### Training

The INT group increased the weekly training volume at HR_zone2_ by 32 ± 22 min and at HR_zone3_ by 55 ± 17 min from the preparatory to the training period, whereas the VOL group increased training volume by 68% ± 5% and running distances by 76% ± 25% (Table 2). Both groups performed lower training volume (*P* < 0.01) compared with the preparatory period during the first (INT = 2.9 ± 1.1, VOL = 2.7 ± 1.2 h) and the second recovery weeks (INT = 2.9 ± 1.1 h, VOL = 2.9 ± 1.5 h), and only LIT, except for the 3000-m running test, was reported during the recovery weeks.

**TABLE 2:** Mean ± SD average weekly training characteristics during the 2-wk preparatory and the 2-wk training periods of high-intensity (HIIT block) or low-intensity training (LIT block).

	INT (*n* = 15)		VOL (*n* = 15)	
	Preparatory	HIIT Block	Preparatory	LIT Block
Training volume (h)	5.8 ± 1.7	5.2 ± 1.1	5.4 ± 2.1	9.0 ± 3.4**
Training frequency per week	5.6 ± 1.4	5.8 ± 1.3	5.3 ± 1.9	5.9 ± 1.8*
Running volume (km)	45.8 ± 12.6	49.8 ± 9.3	44.6 ± 14.7	77.0 ± 22.7**
HR_zone1_ (%)	91.5 ± 5.7	61.9 ± 7.0**	92.2 ± 3.9	99.6 ± 0.7**
HR_zone2_ (%)	6.3 ± 4.7	18.4 ± 6.0**	5.4 ± 2.9	0.4 ± 0.7**
HR_zone3_ (%)	2.2 ± 2.6	19.7 ± 5.2**	2.4 ± 1.7	0.0 ± 0.0**

*P < 0.05, **P < 0.01 compared with the preparatory period.

INT, intensity group; VOL, volume group; HR_zone1_, HR below the first lactate threshold; HR_zone2_, HR between the first and the second lactate threshold; HR_zone3_, HR above the second lactate threshold.

**FIGURE 1:**
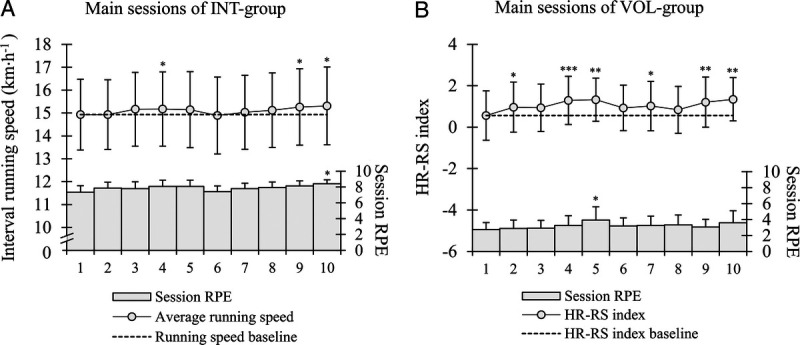
A, Mean ± SD average running speed during the 6 × 3-min intervals performed at maximal sustainable effort, and session RPE of each interval session prescribed. B, Mean ± SD HR-RS index and session RPE of basic (1–4,6–9) and long-distance (5,10) LIT sessions prescribed. *P < 0.05, **P < 0.01, ***P < 0.001 compared with the first session.

Performance and session RPE values of all main sessions are presented in Figure 1. In the VOL group, average running speed and distance covered were 9.8 ± 1.5·h^−1^ and 13.5 ± 3.0 km in the basic sessions and 9.1 ± 1.5 km·h^−1^ and 21.0 ± 4.7 km in the long-distance sessions, respectively. In the INT group, the average HR during the intervals decreased (*P* < 0.05) from the first session (90.7% ± 1.8% HR_max_) to the 6th, 7th, 9th, and 10th sessions (88.1%–88.6% HR_max_). In the VOL group, the average HR remained similar within-session type and was on average 72.6% ± 4.9% HR_max_ during the basic sessions and 69.0% ± 4.5% HR_max_ during the long-distance sessions.

### Physical performance

**FIGURE 2:**
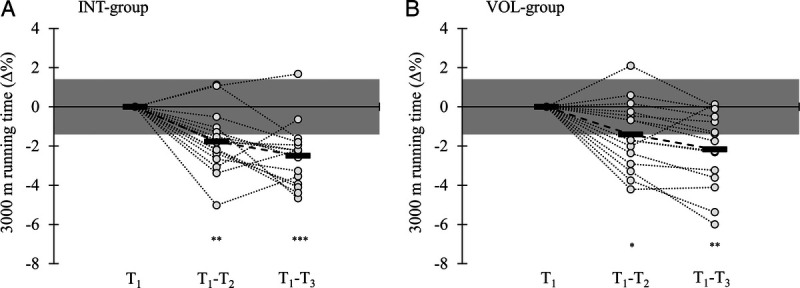
Relative individual (*white plots*) and mean changes (*black rectangle*) in the 3000-m running time immediately after the 2-wk training period (T_1_–T_2_) and after a recovery week (T_1_–T_3_). The *gray* area represents the smallest worthwhile change area (±1.41%), which was the coefficient of variation between T_0_ and T_1_ tests. *P < 0.05, **P < 0.01, ***P < 0.001 compared with T_1_.

A significant main effect of time (*P* < 0.001) was observed in the 3000-m running time as well as HR_avg_ (*P* = 0.004) and HR_peak_ (*P* < 0.001) measured during the test (Table 3). In addition, a significant time–group interaction (*P* < 0.001) was found in HR_avg_ and HR_peak_. Both groups improved the 3000-m running time from T_1_ to T_2_ (INT, *P* = 0.003; VOL, *P* = 0.017) and from T_1_ to T_3_ (INT, *P* < 0.001; VOL, *P* = 0.001) (Fig. 2). No significant main effects nor interaction was observed in the CMJ performance, which was tested before the 3000-m tests (Table 3) or in the tests that were performed before the supervised sessions during the training period (INT, lowest mean = 31.9 ± 5.5 vs highest mean = 32.4 ± 5.1 cm; VOL, lowest mean = 31.0 ± 5.8 vs highest mean = 31.6 ± 6.0 cm).

**TABLE 3:** Mean ± SD average performance test results before the 2-wk training period (T_1_), immediately after the training period (T_2_), and after a recovery week (T_3_).

	INT (*n* = 15)			VOL (*n* = 15)		
	T_1_	T_2_	T_3_	T_1_	T_2_	T_3_
3000 m (min:s)	12:19 ± 1:32	12:06 ± 1:32**, ES = −0.14	12:00 ± 1:27***, ES = −0.21	12:33 ± 1:33	12:22 ± 1:30*, ES = −0.12	12:16 ± 1:29**, ES = −0.18
HR_avg_ (%/max)	94.3 ± 2.4	92.2 ± 2.6***,^##^, ES = −0.85	93.8 ± 2.2^#,*a*^, ES = −0.24	94.7 ± 2.1	94.9 ± 2.1, ES = 0.08	95.3 ± 2.2, ES = 0.33
HR_peak_ (%/max)	99.4 ± 1.9	96.6 ± 2.4***,^###^, ES = −1.29	98.1 ± 1.7**,^##,*a*^, ES = −0.71	98.9 ± 2.3	99.9 ± 2.6, ES = 0.40	99.9 ± 2.3, ES = 0.45
CMJ (cm)	33.0 ± 6.2	32.6 ± 5.6, ES = −0.07	33.5 ± 5.5, ES = 0.09	32.6 ± 6.4	33.1 ± 5.9, ES = 0.07	33.1 ± 6.1, ES = 0.08

*P < 0.05, **P < 0.01, ***P < 0.001 within-group changes compared with T_1_.

^#^*P* < 0.05, ^##^*P* < 0.01, ^###^*P* < 0.001 between-group changes compared with T_1_.

*^a^*Difference observed from T_1_ to T_2_ and T_2_ to T_3_.

INT, intensity group; VOL, volume group; HR_avg_, average HR of the 3000-m running test in relation to the maximum HR of the incremental treadmill test; HR_peak_, peak HR of the 3000-m running test in relation to the maximum HR of the incremental treadmill test; CMJ, countermovement jump test; ES, effect size of the changes from T_1_.

### Physiological responses

A significant main effect of time was observed in hemoglobin (*P* < 0.001), hematocrit (*P* = 0.001), and norepinephrine (*P* < 0.001) (Table 4). In addition, a significant increase was observed in CK activity of VOL from T_1_ to T_2_ (*P* = 0.036). Norepinephrine increased in INT from T_1_ to T_2_ (*P* = 0.01) and remained elevated in T_3_ (*P* = 0.018). Hemoglobin concentration (*P* = 0.011) and hematocrit (*P* = 0.037) decreased from T_1_ to T_2_ in VOL, whereas hemoglobin tended to decrease from T_1_ to T_2_ (*P* = 0.065) and increased from T_2_ to T_3_ (*P* = 0.029) in the INT group. When plasma volume changes were estimated based on hemoglobin and hematocrit values, T_1_–T_2_ changes translated to 4.3% ± 5.0% and 5.1% ± 6.7% expansion in the plasma volume of INT and VOL, respectively.

**TABLE 4:** Mean ± SD average blood and urine sample results before the 2-wk training period (T_1_), immediately after the training period (T_2_), and after a recovery week (T_3_).

	INT			VOL		
	T_1_	T_2_	T_3_	T_1_	T_2_	T_3_
Cor (nmol·L^−1^)	422 ± 88	419 ± 80, ES = −0.03	442 ± 115, ES = 0.20	410 ± 106	459 ± 88, ES = 0.51	465 ± 111, ES = 0.51
Ftesto (pmol·L^−1^)	40.4 ± 27.2	40.6 ± 26.0, ES = 0.00	42.9 ± 28.1, ES = 0.02	35.7 ± 23.3	36.0 ± 26.0, ES = 0.00	39.5 ± 26.2, ES = 0.04
CK (μmol·L^−1^)	103 ± 64	124 ± 53, ES = 0.09	122 ± 130, ES = 0.08	107 ± 35	178 ± 102*, ES = 0.52	126 ± 78, ES = 0.13
Hb (g·L^−1^)	140 ± 9	136 ± 10, ES = −0.37	140 ± 9*^,*a*^, ES = 0.03	145 ± 12	141 ± 11*, ES = −0.36	143 ± 13, ES = −0.16
Hct (%)	42.3 ± 2.7	41.4 ± 3.0, ES = −0.33	42.7 ± 2.8, ES = 0.13	43.8 ± 3.1	42.8 ± 2.7*, ES = −0.35	43.3 ± 2.9, ES = −0.18
NE (μmol)	0.11 ± 0.04	0.15 ± 0.04*, ES = 0.91	0.15 ± 0.04*, ES = 1.03	0.12 ± 0.05	0.13 ± 0.05, ES = 0.19	0.15 ± 0.06, ES = 0.53

*P < 0.05, **P < 0.01 compared with T_1_.

*^a^*Difference observed from T_2_ to T_3_.

INT, intensity group; VOL, volume group; Cor, serum cortisol concentration; Ftesto, serum-free testosterone concentration; CK, serum creatine kinase activity; Hb, hemoglobin concentration; Hct, hematocrit fraction; NE, urine norepinephrine concentration; ES, the effect size of the changes from T_1_.

**FIGURE 3:**
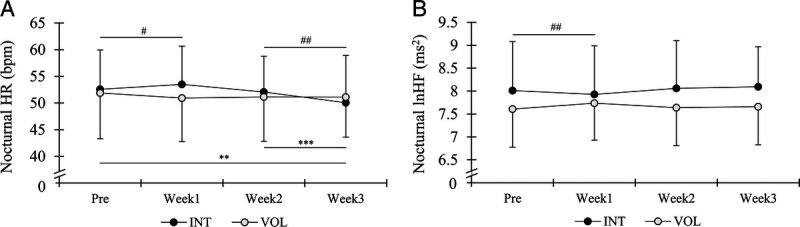
Mean ± SD average weekly nocturnal HR (A) and lnHF (B) at baseline (Pre), during the training period (Week1 and Week2), and recovery week (Week3). INT, interval group; VOL, volume group. **P < 0.01, ***P < 0.001 compared with respective time points in INT. ^#^*P* < 0.05, ^##^*P* < 0.01, between-group changes compared with respective time points.

A significant main effect of time (*P* = 0.001) was found in nocturnal HR, and a significant time–group interaction was found in nocturnal HR (*P* = 0.001), nocturnal lnHF (*P* = 0.036) (Fig. 3), and nocturnal lnRMSSD (*P* = 0.027). Nocturnal HR decreased in INT from Pre to Week3 (*P* = 0.002, ES = −0.36) and from Week2 to Week3 (*P* < 0.001, ES = −0.30). Changes in HR from Pre to Week1 (INT = 1.9% ± 4.0% vs VOL = −1.6% ± 5.1%, *P* = 0.045) and from Week2 to Week3 (INT = −3.8% ± 3.2% vs VOL = 0.1 ± 2.9, *P* = 0.003) were different between the groups. In lnHF, no significant within-group changes were found, but change from Pre to Week1 was different between the groups (INT = −1.0% ± 2.0% vs VOL = 1.8% ± 3.2%, *P* = 0.008). The same pattern was observed in lnRMSSD, which remained unaffected through the training and recovery weeks in INT (4.18 ± 0.52 ms vs 4.14 ± 0.50, 4.21 ± 0.48 and 4.24 ± 0.42 ms) and VOL (4.03 ± 0.43 ms vs 4.10 ± 0.40, 4.06 ± 0.42 and 4.05 ± 0.41 ms), but change from Pre to Week1 differed between the groups (*P* = 0.014).

### Perceptual responses

**FIGURE 4:**
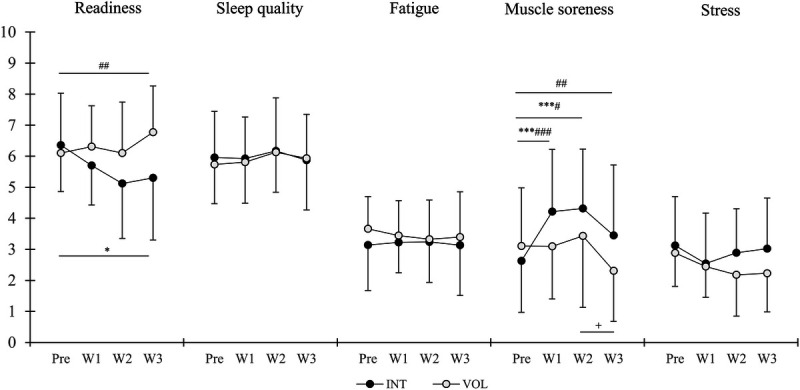
Mean ± SD average weekly values of perceptual recovery at baseline (Pre), during the training period (W1 and W2), and recovery week (W3). INT, interval group; VOL, volume group. *P < 0.05, **P < 0.01, ***P < 0.001 compared with Pre in INT. ^#^*P* < 0.05^, ##^*P* < 0.01, ^###^*P* < 0.001 between-group changes compared with Pre. ^+^Compared with the previous week in VOL.

A significant main effect of time was found in muscle soreness (*P* < 0.001), and a significant time–group interaction was found in the readiness to train (*P* = 0.008) and muscle soreness (*P* = 0.001) (Fig. 4). Readiness to train decreased in INT from Pre to Week3 (*P* = 0.045, ES = −0.57) and tended to decrease from Pre to Week2 (*P* = 0.057, ES = −0.72). In addition, the change in readiness to train from Pre to Week3 was different between the groups (*P* = 0.002). Muscle soreness increased in INT (*P* < 0.001) from Pre to Week1 (ES = 0.86) and Week2 (ES = 0.94), and the change was different between the groups from Pre to Week1 (*P* < 0.001), Week2 (*P* = 0.012), and Week3 (*P* = 0.001).

### Relationships between monitoring variables and changes in endurance performance

A significant positive correlation was found between the relative change in average running speed from 1st to 10th interval session and relative change in the 3000-m running speed from T_1_ to T_2_ in INT (*r* = 0.656, *P* = 0.008). In VOL, a tendency for negative correlation was found between the change in HR-RS index from 1st to 10th low-intensity session and relative change in the 3000-m running speed from T_1_ to T_2_ (*r* = −0.510, *P* = 0.052). In addition, the relative change in the nocturnal HR from Pre to Week2 correlated positively with the relative change in the 3000-m running speed from T_1_ to T_3_ in VOL (*r* = 0.538, *P* = 0.047). Among the perceptual markers and INT group, muscle soreness at Week2 correlated negatively (*r* = −0.564, *P* = 0.028), and change in the readiness to train from Pre to Week2 correlated positively (*r* = 0.529, *P* = 0.043) with the relative change in the 3000-m running speed from T_1_ to T_2_. The change in the stress from Pre to Week2 was the only marker that correlated significantly with the relative change in the 3000-m running speed from T_1_ to T_3_ in INT (*r* = 0.637, *P* = 0.011). When groups were pooled, fatigue (*r* = −0.449, *P* = 0.013) and muscle soreness (*r* = −0.375, *P* = 0.041) at Week2 both correlated negatively with the relative change in the 3000-m running speed from T_1_ to T_2_. Full results of all correlation analyses are presented in Supplementary Table 1 (see Table, Supplemental Digital Content 1, Pearson correlation coefficient between monitoring variables and relative change in the 3000-m running speed, http://links.lww.com/MSS/C488).

## DISCUSSION

The main findings of the study were that 2-wk blocks of HIIT or LIT both improved the 3000-m running performance, and no differences were found between the groups in the training adaptations. Based on physiological and perceptual responses during the blocks, both periods could be tolerable for recreational athletes, although the HIIT block induced some negative responses compared with the LIT block, such as increased muscle soreness and decreased HRV. Running speed during the interval sessions and resting-state perceptual recovery seemed to be useful monitoring tools for acute responses to intensified training blocks.

### Training and performance

HIIT microcycles have previously been examined mainly by 1- to 3-wk periods of >4 HIIT sessions (4), whereas typical volume periods have increased training volume by 30%–100% for 2–6 wk (6–9). The current protocols were chosen to produce a significant but tolerable increase in the training load via either training intensity or training volume, but not at the same time. The 3000-m running performance improved in both groups already at T_2_, but no significant differences were found after the recovery week between T_2_ and T_3_. Therefore, the training load seemed to be suitable on average, and neither of the blocks induced significant acute fatigue at group level. It has been suggested that LIT training would more likely lead to positive (30) or very positive (31) training adaptations compared with HIT training. The present results did not support these findings, at least among the block periodization, as peak performance improved more than the coefficient of variation of the 3000-m test out of 14/15 participants in the INT and 9/15 in the VOL groups. Although both of the current 2-wk block protocols induced significant improvements in endurance performance, previous studies suggest that a combination of HIT and LIT may be needed for the optimal long-term development of endurance capacity (1,3).

After overload protocols, positive training adaptation is typically delayed because of acute fatigue or overreaching effect (5). Previously, after the high-volume 3-wk overload period, peak performance has been obtained after a 2-wk taper (9). After a high-frequency 3-wk HIIT period, the peak performance was achieved after 12 d (32). From this perspective, it was interesting that 4/15 participants of the INT group impaired their running performance after the recovery week, whereas there was only one clear impairment in the VOL group. This could partially relate to tapering, which included no HIIT sessions. Although the intensity is suggested to be maintained in optimal tapering (33), no HIIT sessions were prescribed to allow a similar recovery week for both groups in the present study. Therefore, it may be possible that some individuals have experienced some type of detraining effect after the low-volume and low-intensity recovery week.

Although the performance improved similarly in both groups immediately after the training period, peak and average HR during the running test decreased only in the INT group, and peak HR remained decreased at T_3_. This may relate to decreased activity of the sympathetic nervous system via a reduced adrenergic response during exercise (21) or the down-regulation of β-adrenoreceptors (34) due to repetitive training at high intensity. A similar trend was observed during the intervals, where average HR decreased, especially during the second week of the training period, despite maintained or even increased running speed. It would be interesting to know whether the decrement was compensated with improved cardiac stroke volume, which has occurred after various HIIT protocols (32,35). Based on previous studies of volume overloads (6,8), it was expected that the LIT block would also decrease HR in the 3000-m tests, possibly by increased blood volume (36) and parasympathetic hyperactivity (8). Lack of changes in the VOL group could partially be related to the lower absolute training volumes of recreational athletes compared with previous studies of well-trained athletes (6,8).

### Physiological and perceptual responses to training

Although studies targeting overload may provide information regarding the state of overreaching, the effects of the increased volume or intensity *per se* seem to remain unsolved. It is possible that physiological responses to intensified training periods have varied depending on the method of increasing training load. In the current study, the only significant change in hormonal markers was found in the resting norepinephrine concentration, which increased in INT and remained elevated also after the recovery period. The finding somewhat contradicted previous studies, which have shown that resting values either remained similar (7,21) or decreased after intensified training (6). Based on the increase in resting norepinephrine and the decrease in peak HR during the 3000-m test after a demanding block of frequent HIIT training, a longer recovery period may be advisable to restore normal autonomic nervous system function at rest and during exercise. From the other biomarkers, creatine kinase increased in VOL at T_2,_ whereas it was anticipated that HIIT block would also increase CK concentration (37,38). It is possible that the higher training volume of VOL, as well as a long-distance session 2 d before the CK assessment, may have induced more structural damage in the musculoskeletal tissue compared with HIIT. Previously, Quinn and Manley (39) observed elevated CK values even 72 h after a 26-km run at 60%–75% HR_max_, which was similar to the long-distance session of the VOL group performed in the present study.

Resting HRV is a marker used to analyze the restoration of cardiovascular homeostasis and the stress/recovery state in general (40). Although increments in HRV are typically a positive sign of the increased parasympathetic activity and a good state of recovery (40), the so-called parasympathetic hyperactivity is an abnormal response to a sudden increase in training load (8,19). Previously, overload microcycles have induced significant increases in HRV with the concurrent increase of fatigue (8,19). In the current study, no significant changes in HRV were found, although the response to the first week differed between the groups (a slight decrease in INT vs an increase in VOL). It seems that the parasympathetic hyperactivity may be related to the overreaching/fatigue state itself rather than to the increased training load, as fatigue was not increased at the group level in the current study. The HRV response to the increased training load, an increase or decrease, seems to be individual, despite the type of training (25). Interestingly, nocturnal HR decreased significantly during the recovery period in the INT group, with no changes at any week in VOL. Similar findings have been observed previously (25), suggesting that high-intensity training may induce different cardiac adaptations compared with high-volume training. Although resting HR or HRV and catecholamine concentration are thought to reflect the autonomic nervous system function from another perspective, it seems that responses to intensified training may differ between these markers.

Although physiological markers provide objective information about the biological processes, perceptual markers may also provide valuable information to predict maladaptation to training (13,14) and help contextualize changes in the physiological markers (8,19). In the present study, the most significant changes were found in muscle soreness, which increased in INT and differed from changes in VOL along all the training and recovery weeks. Interestingly, the result somewhat contradicted the result of CK, which increased only in VOL. Concerning possible explanations, HIIT running differs from LIT from a biomechanical point of view (cadence, ground reaction forces) (41), and HIIT would most likely induce more strain in the type II motor units compared with LIT (1). Altogether, relative unfamiliarity combined with the abnormally high HIT frequency may have increased muscle soreness locally in the running muscles. Furthermore, CK may be elevated without an increase in muscle soreness after low-intensity running (39).

### Relationships between monitoring variables and changes in running performance

Because responses to endurance training periods are quite individual, it may be challenging to find unambiguous connections between monitoring variables and changes in performance (25). In the current study, there were associations among several markers of subjective recovery (fatigue, muscle soreness, readiness to train, and stress) and changes in running performance. Previous studies have also shown that subjective markers, such as fatigue and readiness to train (14), or the sum of multiple well-being ratings (13) may be useful indicators in the prediction of overreaching or overtraining. Therefore, maintaining these parameters within the normal range seems desirable during intensified training periods. Interestingly, change in stress from Pre to Week2 was positively associated with the final training adaptation in INT. Ruuska et al. (42) have previously found that mental stress may impair training adaptation to endurance training, and it is generally suggested that intensive training may not be recommended during periods of increased stress. Because absolute stress values remained rather low through the training block, the current association was most likely coincidence, and it highlights the importance of reliable reference values when assessing individual responses in the subjective monitoring variables.

Another interesting finding was that the change in running speed from the 1st to the 10th interval session correlated with the change in the 3000-m running speed from T_1_ to T_2_. However, the same change did not correlate with the change from T_1_ to T_3_. Therefore, maximal sustainable running speed during the intervals seemed to represent the current performance level, rather than predicting the final training adaptation after a sufficient recovery period. In the VOL group, a similar but negative tendency was found between the change in the HR-RS index and the change in running speed from T_1_ to T_2_. Although the HR-RS index may be a useful tool in the long-term monitoring of training adaptation (23,25), in this type of short-term blocks where submaximal HR tend to drop, it or other HR-based markers should be used in accordance with perceptual markers (8,19).

### Limitations

In the current study, responses to HIIT and LIT blocks were examined in males and females, but the number of participants did not, unfortunately, allow true comparisons between the sexes. Participants of the present study were recreationally trained, meaning these results should not be extrapolated uncritically to either untrained or well-trained athletes. Changes in endurance performance were assessed only with the 3000-m running test, so we cannot identify specific physiological adaptations underpinning the measured performance improvements.

## CONCLUSIONS

In conclusion, both the 2-wk block of HIIT and LIT elicited statistically significant and practically meaningful short-term performance improvements. Based on the responses observed in the monitoring variables, both blocks seemed tolerable for recreational athletes. However, the HIIT block induced some negative responses not observed in response to a comparable VOL overload. This may indicate higher demands of training compared with LIT and less “margin for error” when designing this block training intervention in practice. Ensuring sufficient recovery especially after such a period would therefore be of importance. Monitoring subjective recovery alongside performance and objective markers may provide the most valid and actionable assessment of current “readiness to train.”
